# Influence of procedural differences on mitral valve configuration after surgical repair for functional mitral regurgitation: in which direction should the papillary muscle be relocated?

**DOI:** 10.1186/s13019-014-0185-6

**Published:** 2014-12-10

**Authors:** Taiju Watanabe, Hirokuni Arai, Eiki Nagaoka, Keiji Oi, Tsuyoshi Hachimaru, Hidehito Kuroki, Tatsuki Fujiwara, Tomohiro Mizuno

**Affiliations:** The Department of Cardiovascular Surgery, Tokyo Medical and Dental University Graduate School of Medical and Dental Sciences, 1-5-45 Yushima, Bunkyo-ku, Tokyo, Japan

**Keywords:** Functional mitral regurgitation, Tethering, Mitral valve repair, Papillary muscle relocation

## Abstract

**Background:**

After restrictive mitral annuloplasty (RMAP) for functional mitral regurgitation (MR), the MR frequently recurs. Papillary muscle relocation (PMR) should reduce the recurrence rate. We assessed the influence of procedural differences in PMR on the postoperative mitral valve configuration.

**Methods:**

Thirty-nine patients who underwent mitral valve repair for functional MR were enrolled. In limited tethering cases, RMAP alone was performed (RMAP group; n = 23). In severe tethering cases, in addition to RMAP, bilateral papillary muscles were relocated in the direction of the posterior annulus (posterior PMR group; n = 10) or anterior annulus (anterior PMR group; n = 6). We performed pre- and postoperative transthoracic echocardiographic studies, introducing a new index, mitral inflow angle (MIA), to assess the diastolic mitral leaflet excursion. MIA was measured as the angle between the mitral annular plane and the bisector of the anterior and posterior leaflets.

**Results:**

Postoperative MR grade was significantly reduced in each group (P < 0.001). Follow-up echocardiography showed recurrent MR in 13% of the patients in RMAP group. In contrast, no recurrent MR was observed in either the anterior PMR or the posterior PMR group. After surgery, MIA was significantly reduced in both the RMAP group (P < 0.01) and the posterior PMR group (P < 0.001), but was preserved in the anterior PMR group (NS). None of the postoperative variables showed any significant difference between the early and late postoperative phases.

**Conclusions:**

In the surgical treatment of functional MR, a PMR procedure in addition to RMAP was effective in reducing systolic MR. However, mitral valve opening assessed by MIA was restricted even after RMAP alone. The restriction was severely augmented after additional posterior PMR, but was attenuated after additional anterior PMR. The papillary muscle should be relocated in the direction of the anterior annulus to preserve the diastolic opening of the mitral valve.

**Electronic supplementary material:**

The online version of this article (doi:10.1186/s13019-014-0185-6) contains supplementary material, which is available to authorized users.

## Background

Functional mitral regurgitation (MR) remains one of the most complex and unresolved entities in the management of heart valve disease [1]. If left untreated, functional MR is associated with an increase in mortality [2],[3]. Currently, there is general agreement about the efficacy of surgical treatment for patients with severe functional MR, but there are differing opinions as to the best surgical approach [4]. Restrictive mitral annuloplasty (RMAP), which was first introduced by Bolling and colleagues, has become a standard procedure for treating functional MR [5]. However, this therapeutic approach has been associated with a high recurrence rate of functional MR, reaching as much as 30% [6],[7]. Many surgeons favor adding subvalvular procedures to RMAP as a means of reducing the tethering forces and improving the long-term results.

As an adjunct to mitral annuloplasty, Kron and colleagues developed a procedure for relocating the posterior papillary muscle toward the mitral annular plane. In this technique, a polypropylene suture is passed through the fibrous portion of the posterior papillary muscle and then passed up through the adjacent mitral annulus posterior to the right fibrous trigone. The posterior papillary muscle is subsequently relocated to the point at which leaflet coaptation occurs in the plane of the mitral annulus [8]. Papillary muscle relocation (PMR) could be expected to relieve mitral valve tethering and to reduce the recurrence rate of MR [9],[10], but its effectiveness in practice has not been established.

In an early series of patients undergoing surgical treatment for severe functional MR, we performed bilateral PMR in the direction of the posterior annulus in addition to RMAP [11]. Postoperative echocardiography demonstrated successful treatment as regards mitral valve function during systole. However, during diastole, the anterior mitral leaflet excursion was restricted and there was a mosaic pattern in the Doppler color flow mapping of the transmitral flow, which reflected a restriction of mitral inflow. Therefore, in recent years, we changed the PMR direction from posterior annulus to anterior annulus, with a view to achieving more physiological mitral valve excursion and hence better diastolic mitral valve inflow. In this study, we used echocardiography to investigate the influence of these procedural differences on the postoperative mitral valve configuration and to determine the optimal direction of PMR in order to achieve the best possible mitral valve function.

## Methods

### Patients

Thirty-nine patients who underwent mitral valve repair for functional MR between January 2005 and December 2012 were enrolled in the study. These included 32 cases of ischemic functional MR and 7 cases of non-ischemic functional MR. All patients with functional MR had restrictive systolic leaflet motion (Carpentier type IIIb). The exclusion criteria were organic mitral valve lesion (rheumatic, infective, degenerative), concomitant aortic valve surgery, and ventricular assist device implantation. Prior to surgery, all patients had been treated with optimal medication, including angiotensin-converting enzyme inhibitors or angiotensin receptor blockers, β-blockers, and diuretics. The study was approved by Institutional Review Board of Tokyo Medical and Dental University, and written informed consent was obtained from all patients.

Our surgical strategy for functional MR is that patients who exhibit severe tethering, in which a coaptation depth of ≥1.0 cm [12] and a posterior leaflet angle at mid-systole of ≥45° [13] are measured in the preoperative echocardiographic study, are treated by additional PMR in the direction of the anterior or posterior annulus, whereas patients with limited tethering are treated by RMAP alone.

Following this strategy, RMAP alone was performed in 23 patients (RMAP group) and RMAP with PMR was performed in 16 patients. Among the 16 patients who underwent additional PMR, the bilateral papillary muscles were relocated in the direction of the posterior annulus in 10 patients (posterior PMR group) and the anterior annulus in 6 patients (anterior PMR group).

### Echocardiographic measurements

All patients were examined by standard two-dimensional and Doppler transthoracic echocardiography within 1 week before operation (preoperative), 1–2 weeks after operation (postoperative), and more than 3 months after operation (follow-up). Intraoperative transesophageal echocardiography was used in all patients to evaluate the quality of repair. All examinations were performed by independent experienced echocardiographers.

The left ventricular (LV) end-diastolic and end-systolic diameters were measured using M-mode in the parasternal long-axis view. The LV ejection fraction was determined by the modified Simpson method. In the present study, we focused on mitral valve configuration and function during systole and diastole.

#### Systolic mitral leaflet closure

**Figure 1:**
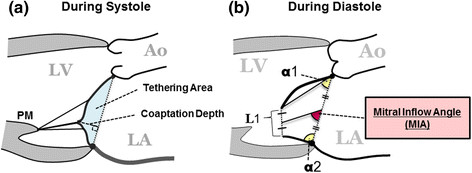
**Assessment of mitral valve configuration during systole (a) and diastole (b).** MIA: the angle between the mitral annular plane and the bisector of the anterior and posterior leaflets; α1: the opening angle between the anterior leaflet and the mitral annular plane; α2: the opening angle between the posterior leaflet and the mitral annular plane; L1: the distance between the open leaflet tips; Ao: aorta; LV: left ventricle; LA: left atrium; PM: papillary muscle.

To assess systolic mitral leaflet closure, MR grade, coaptation depth, and tethering area were evaluated (Figure 1a). MR grade was quantified as none (0), mild (1+), moderate (2+), moderate-to-severe (3+), or severe (4+), based on the regurgitant color jet shape and area in relation to the left atrial area. Coaptation depth was defined as the distance between the mitral annular plane and the coaptation point of the mitral leaflets, and tethering area was defined as the area formed by the mitral annular plane and both mitral leaflets in the parasternal long-axis view at the time of maximal systolic closure.

#### Diastolic mitral leaflet excursion

**Figure 2:**
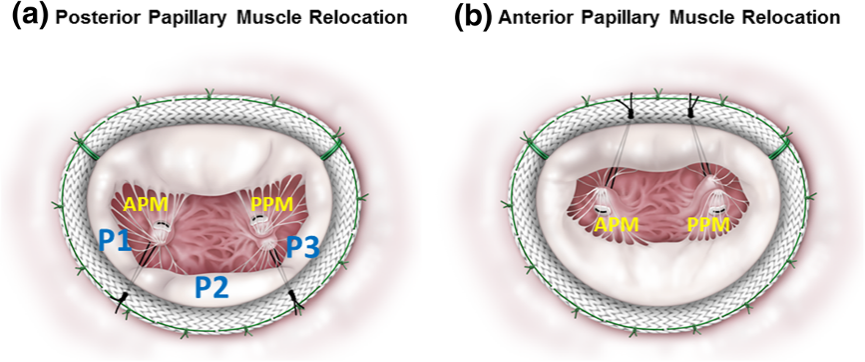
**Surgical technique for papillary muscle relocation (PMR). (a)** In posterior PMR, each pair of polytetrafluoroethylene sutures was passed through the posterior annulus at the corresponding border of the lateral (P1) and middle (P2) segments of the posterior annulus, or at the border of the middle (P2) and medial (P3) segments. **(b)** In anterior PMR, both pairs of sutures were passed through the mid-anterior annulus. APM: anterior papillary muscle; PPM: posterior papillary muscle; P1: lateral segment of the posterior annulus; P2: middle segment of the posterior annulus; P3: medial segment of the posterior annulus.

To assess diastolic mitral leaflet excursion, 4 parameters, α1, α2, L1, and mitral inflow angle (MIA), were measured in the parasternal long-axis view at the time of maximal leaflet opening (Figure 2b). The first parameter, α1, is the opening angle between the anterior leaflet and the mitral annular plane, while α2 is the opening angle of the posterior leaflet. L1 is the distance between the tips of the open leaflets. The new index, MIA was introduced to assess the diastolic opening of the mitral valve in a comprehensive way. MIA was defined as the angle between the mitral annular plane and the bisector of the anterior and posterior leaflets.

### Surgical technique

The mitral valve was exposed through a right-sided left atriotomy. U-shaped 2-0 braided simple horizontal sutures were placed at the annulus to optimize the exposure of the subvalvular apparatus. Polytetrafluoroethylene mattress sutures (CV-4, W. L. Gore & Associates, Flagstaff, AZ) with pledgets of autologous pericardium were placed in the fibrous portions of both the anterior and posterior papillary muscle tips. In the posterior PMR procedure, the free ends of each pair of CV-4 sutures were passed through the posterior annulus at the corresponding border of the lateral (P1) and middle (P2) segments of the posterior annulus, or at the border of the middle (P2) and medial (P3) segments (Figure 2a). In the anterior PMR procedure, the free ends of the CV-4 sutures were passed through the mid-anterior annulus (Figure 2b). The CV-4 sutures were then also passed through the corresponding parts of the semi-rigid annuloplasty ring (Carpentier-Edwards Physio II; Edwards Life sciences, Irvine, CA). Ring size was determined after measurement of the height of the anterior leaflet and then downsized by 2 sizes (mean: 26.4 mm, range: 24–28 mm). The annuloplasty ring was seated and the CV-4 sutures were pulled to relocate the bilateral papillary muscle tips closer to the annulus, at the point at which leaflet coaptation occurred in the mitral annular plane. To determine the optimal length of the relocation sutures during systole, we adjusted the sutures on a beating heart, confirming no residual MR, as described in our previous report [11].

### Statistical analysis

Continuous variables are expressed as mean ± standard deviation and categorical variables as frequencies and percentages. Comparisons among the 3 groups were conducted using one-way analysis of variance (ANOVA), followed by the Tukey–Kramer test for continuous variables and the χ^2^ test for categorical variables. Comparisons of continuous variables over time in each group were made using repeated measures ANOVA followed by a paired t-test with the Bonferroni correction. A P-value <0.05 was defined as statistically significant. Statistical analysis was performed using Statview version 5.0 for windows (SAS Institute, Cary, NC, USA).

## Results

### Baseline characteristics

The patients’ baseline characteristics are listed in Table 1. There was no statistical difference among the 3 groups in preoperative LV end-diastolic diameter. In both anterior PMR and posterior PMR groups, preoperative MR grade was more advanced (P < 0.01), and preoperative LV ejection fraction indicated poorer LV function (P = 0.026) compared with the RMAP group.

**Table 1:** **Baseline characteristics**

	RMAP	Posterior PMR	Anterior PMR	P-value
	(n = 23)	(n = 10)	(n = 6)	
**Age, years**	70 ± 6	65 ± 7	68 ± 8	0.16
**Male**	17 (73.9)	9 (90.0)	3 (50.0)	0.21
**NYHA functional class**				
**1**	2 (8.7)	0	0	0.48
**2**	12 (52.2)	3 (30.0)	1 (16.7)	0.21
**3**	8 (34.8)	4 (40.0)	3 (50.0)	0.79
**4**	1 (4.3)	3 (30.0)	2 (33.3)	0.071
**Preoperative inotropic support**	2 (8.7)	3 (30.0)	2 (33.3)	0.19
**Etiology**				
**Ischemic**	23 (100)	8 (80.0)	1 (16.7)	<0.001
**Non-ischemic**	0	2 (20.0)	5 (83.3)	<0.001
**Echocardiographic data**				
**MR grade**	2.6 ± 0.6	3.8 ± 0.4^a^	3.7 ± 0.5^a^	<0.01
**LVEDD (mm)**	66.3 ± 6.4	70.4 ± 7.4	66.2 ± 3.6	0.24
**LVESD (mm)**	48.7 ± 12.0	61.2 ± 9.7^a^	57.3 ± 4.2	<0.01
**LVEF (%)**	40.4 ± 11.7	31.9 ± 5.4^a^	29.7 ± 7.4^a^	0.026

Data are mean ± standard deviation, or n (%). MR: Mitral regurgitation; RMAP: Restrictive mitral annuloplasty; PMR: Papillary muscle relocation; LVEDD: Left ventricular end-diastolic diameter; LVESD: Left ventricular end-systolic diameter; LVEF: Left ventricular ejection fraction.

^a^P < 0.05 compared with RMAP (Tukey–Kramer test).

### Serial echocardiographic evaluation of mitral valve function during systole and diastole

Serial echocardiographic findings are listed in Table 2. The interval between the operation and the last postoperative follow-up echocardiographic study was 28.5 ± 24.9, 33.2 ± 29.1, and 27.3 ± 27.8 months in the RMAP, posterior PMR, and anterior PMR groups, respectively (NS).

**Table 2:** **Serial echocardiographic findings of mitral valve function during systole and diastole**

	RMAP	Posterior PMR	Anterior PMR	P-value
**Preoperative**				
**MR grade**	2.6 ± 0.6	3.8 ± 0.4^a^	3.7 ± 0.5^a^	<0.01
**CD (cm)**	0.8 ± 0.2	1.4 ± 0.5^a^	1.1 ± 0.1	<0.01
**Tethering area (cm** ^**2**^ **)**	1.5 ± 0.5	2.4 ± 0.6^a^	1.9 ± 0.5	<0.01
**MIA (°)**	68 ± 8	61 ± 6	68 ± 7	0.076
**α1 (°)**	54 ± 10	49 ± 9	55 ± 6	0.32
**α2 (°)**	84 ± 17	83 ± 13	83 ± 9	0.99
**L1 (cm)**	1.8 ± 0.4	1.8 ± 0.4	1.8 ± 0.1	0.98
**Postoperative**				
**MR grade**	0.4 ± 0.6**	0.5 ± 0.5**	0.5 ± 0.5**	0.25
**CD (cm)**	0.4 ± 0.2**	0.4 ± 0.1**	0.5 ± 0.3**	0.59
**Tethering area (cm** ^**2**^ **)**	0.6 ± 0.5*	0.4 ± 0.2*	0.7 ± 0.4*	0.41
**MIA (°)**	60 ± 9*	42 ± 10^a,c,^**	67 ± 6	<0.01
**α1 (°)**	55 ± 12	39 ± 9^a,c,^*	67 ± 12*	<0.01
**α2 (°)**	100 ± 12*	106 ± 22*	85 ± 10^b^	0.045
**L1 (cm)**	1.4 ± 0.3*	1.1 ± 0.2^a,^*	1.3 ± 0.1*	0.012
**Follow-up**				
**MR grade**	0.6 ± 0.6**	0.7 ± 0.4**	0.5 ± 0.4**	0.62
**CD (cm)**	0.5 ± 0.2**	0.5 ± 0.3**	0.5 ± 0.3**	0.74
**Tethering area (cm** ^**2**^ **)**	0.8 ± 0.5*	0.5 ± 0.3*	0.7 ± 0.4*	0.21
**MIA (°)**	62 ± 9*	43 ± 10^a,c,^**	69 ± 4	<0.01
**α1 (°)**	55 ± 11	40 ± 9^a,c,^*	67 ± 15*	<0.01
**α2 (°)**	101 ± 11*	107 ± 16*	85 ± 12^a,b^	0.013
**L1 (cm)**	1.4 ± 0.3*	1.1 ± 0.1^a,^*	1.3 ± 0.1*	0.017

Data are mean ± SD, or n (%). MR: Mitral regurgitation; RMAP: Restrictive; mitral annuloplasty; PMR: Papillary muscle relocation; CD: Coaptation depth; MIA (Mitral inflow angle): The angle between the mitral annular plane and the bisector of the anterior and posterior leaflets; α1: Opening angle between the anterior leaflet and the mitral annular plane; α2, Opening angle between the posterior leaflet and the mitral annular plane. L1: Distance between tips of open leaflets.

^a^P < 0.05 compared with RMAP, ^b^P < 0.05 compared with posterior PMR, ^c^P < 0.05 compared with anterior PMR (Tukey–Kramer test). *P < 0.01 compared with preoperative value, **P < 0.001 compared with preoperative value.

#### Systolic mitral leaflet closure

**Figure 3:**
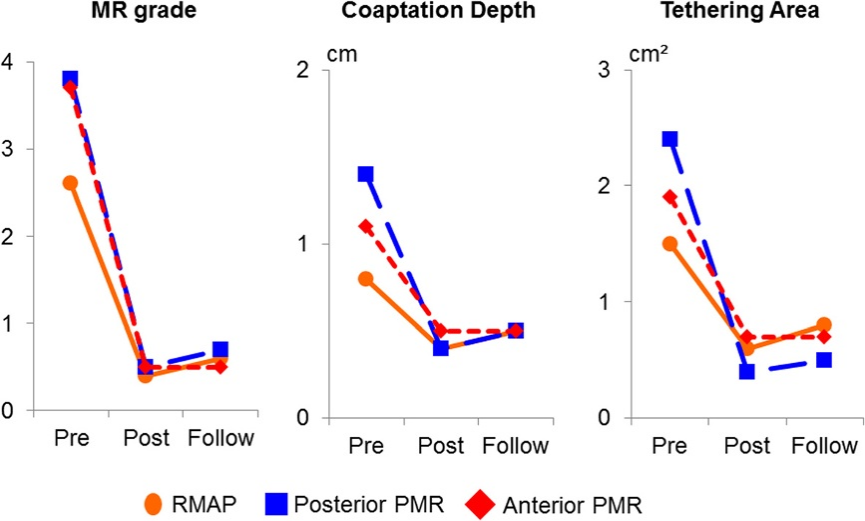
**Recurrence of mitral regurgitation (MR) after repair.** Pre: preoperative phase; Post: early postoperative phase; Follow: late postoperative phase.

**Figure 4:**
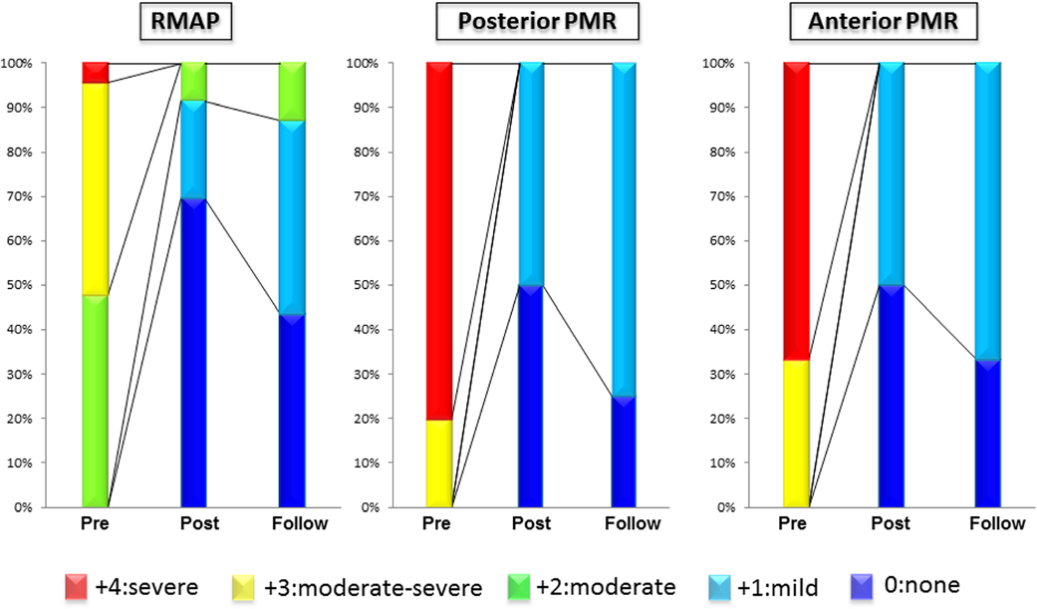
**Serial changes in systolic mitral leaflet closure.** RMAP: restrictive mitral annuloplasty; PMR: papillary muscle relocation.

Preoperative echocardiography showed significant differences in MR grade (P < 0.01), coaptation depth (P < 0.01), and tethering area (P < 0.01) among the 3 groups. Intraoperative post-repair transesophageal echocardiography showed the absence of MR in all patients. In the early and late postoperative phases, all groups showed significant improvements in MR grade (P < 0.001), coaptation depth (P < 0.001), and tethering area (P < 0.01) compared with preoperative values (Figure 3). Postoperative echocardiography showed recurrent MR of grade 2+ or more in 8.7% (n = 2) of the patients in the RMAP group during the early postoperative phase, and in 13% (n = 3) during the late postoperative phase. In contrast, we observed no recurrent MR of grade 2+ or more in either the anterior PMR or the posterior PMR group in the early and late postoperative phases (Figure 4).

#### Diastolic mitral leaflet excursion

**Figure 5:**
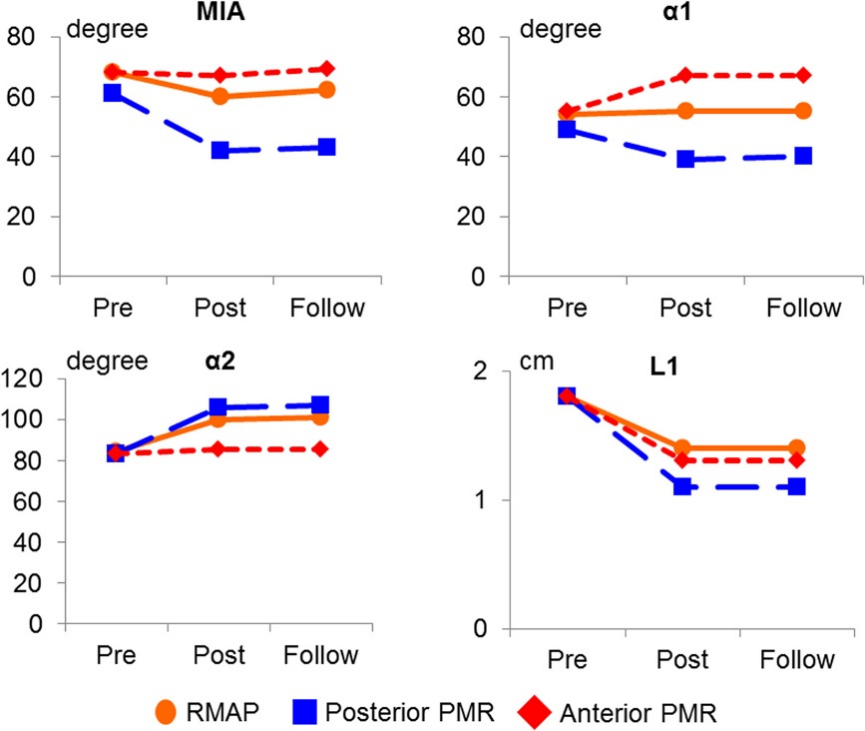
**Serial changes in diastolic mitral leaflet excursion.** Mitral inflow angle (MIA): the angle between the mitral annular plane and the bisector of the anterior and posterior leaflets; α1: the opening angle between the anterior leaflet and the mitral annular plane; α2: the opening angle between the posterior leaflet and the mitral annular plane; L1: the distance between the open leaflet tips.

Preoperatively, the parameters MIA, α1, α2, and L1 showed no significant difference among the 3 groups (Figure 5). After surgery, the opening angle of the anterior leaflet (α1) significantly decreased in the posterior PMR group (P < 0.01), but increased in the anterior PMR group (P < 0.01). The opening angle of the posterior leaflet (α2) significantly increased in the RMAP group (P < 0.01) and the posterior PMR group (P < 0.01), but was preserved in the anterior PMR group (NS). The distance between the open anterior and posterior leaflet tips (L1) decreased in all study groups (P < 0.01). Mitral valve opening assessed by MIA was significantly reduced in both the RMAP group (P < 0.01) and the posterior PMR group (P < 0.001), but was preserved in the anterior PMR group (NS). None of the postoperative variables showed any significant difference between the early and late postoperative phases.

## Discussion

The results of this study support the following conclusions: (1) during systole, each procedure was effective in treating functional MR, while both anterior PMR and posterior PMR procedures were effective in reducing residual/recurrent MR in the high-risk subpopulation of patients with severe tethering; and (2) during diastole, mitral valve excursion, assessed by MIA, was restricted even after the RMAP procedure alone. This restriction was severely augmented after an additional posterior PMR procedure, but was attenuated after additional anterior PMR.

### Surgical strategy for functional mitral regurgitation

The treatment of patients with heart failure whose LV dysfunction is associated with functional MR is challenging. Functional MR is determined by such complex mechanisms as LV remodeling with posterior papillary muscle displacement, leaflet tethering, and mitral annular enlargement, and thus the optimal surgical treatment for functional MR remains controversial [14]. RMAP has recently become the preferred treatment for functional MR, but a considerable number of patients show persistent or recurrent MR despite annuloplasty, which affects their clinical outcome [6],[15],[16]. In the present study, three recurrent MR patients were observed during the postoperative period in the RMAP-only group. These patients showed a tendency to have more dilated preoperative left ventricles (mean LV end-diastolic and end-systolic diameter, 70.6 ± 1.9 mm and 53.8 ± 4.2 mm, respectively) than other patients in the RMAP group without recurrent MR. However, a statistical analysis of the parameters between the recurrent and non-recurrent MR patients was difficult owing to the small numbers of patients in the groups. Preoperative coaptation depth, tethering area, and MIAs were similar between the recurrent and non-recurrent MR patients (data not shown). This suggests that additional subvalvular PMR procedures should be considered when the left ventricle is extremely dilated, even if the extent of the tethering is limited. Currently, several investigators have advocated surgical procedures in addition to RMAP, such as posterior LV plication [17], papillary muscle relocation [8]-[11], papillary muscle approximation [18], LV reconstruction [19],[20], and second chordae cutting [21]. To improve the repair results, we have performed additional PMR procedures in the treatment of patients with severe tethering. In our early series, we relocated the bilateral papillary muscles in the direction of the posterior annulus, resulting in successful repair of severe functional MR during systole [11]. However, we frequently observed restricted mitral valve opening during diastole, which could influence the mitral inflow. While we were developing the PMR procedure, the question arose as to the best direction toward which the papillary muscles should be relocated for more physiological mitral valve excursion.

### Functional mitral stenosis induced by subvalvular tethering

Concerning diastolic mitral valve configuration and function in mitral valve tethering, Otsuji and colleagues reported that patients with LV dysfunction and incomplete systolic mitral leaflet closure also had restricted diastolic leaflet excursion [22]. Other studies showed that RMAP frequently reduced mitral valve opening and caused functional mitral stenosis (MS). Magne and colleagues demonstrated that functional MS induced by RMAP was associated with hemodynamic impairment and pulmonary hypertension [23]. Kubota and colleagues reported that persistent subvalvular diastolic tethering after RMAP procedure induced functional MS at the leaflet tip level and was related to the development of heart failure [24].

### Influence of papillary muscle relocation on mitral valve configuration

**Figure 6:**
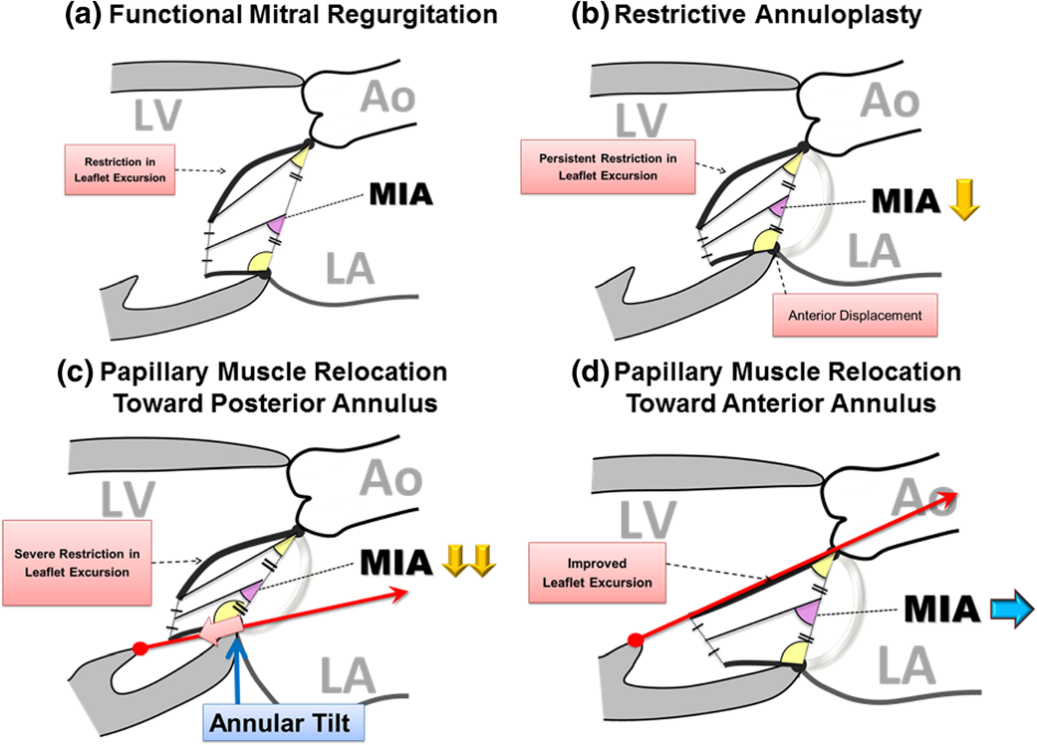
**Influence of procedural differences on diastolic mitral valve configuration. (a)** In patients with functional MR, subvalvular tethering reduces the leaflet mobility even during diastole, because of restriction of the anterior leaflet excursion. **(b)** In the restrictive mitral annuloplasty (RMAP) procedure, posterior leaflet tethering is augmented by the anterior displacement of the posterior mitral annulus, with persistent restriction of the anterior leaflet excursion. This persistent restriction of the anterior leaflet excursion and augmented posterior leaflet tethering diminishes the mitral inflow angle (MIA), changing the inflow direction posteriorly. **(c)** An additional posterior papillary muscle relocation (PMR) procedure (red arrow) not only relocates the papillary muscles, but also pushes down the posterior mitral annulus towards the apex (pink arrow). This induces a mitral annular tilt effect, which involves augmentation of posterior leaflet tethering, diminution of anterior leaflet excursion, decreased leaflet tip opening distance, and comprehensively a severe reduction in MIA, leading to severe impairment of diastolic mitral inflow. **(d)** An additional anterior PMR procedure (red arrow) works not only to compensate for the posterior leaflet tethering induced by RMAP, but also to improve the restricted diastolic excursion of the anterior leaflet. Although the relocation suture may limit the maximal excursion of the anterior leaflet, MIA is well preserved and the diastolic mitral valve configuration is most physiologically maintained among these 3 surgical procedures. Mitral inflow angle (MIA): the angle between the mitral annular plane and the bisector of the anterior and posterior leaflets; Ao: aorta; LV: left ventricle; LA: left atrium.

In the present study, we focused on the influence of procedural differences in PMR on mitral valve configuration and function. During systole, both anterior PMR and posterior PMR procedures were similarly effective in reducing the tethering force and restoring mitral leaflet coaptation in patients with severe tethering. To evaluate the diastolic opening of the mitral valve more comprehensively, we devised a new index, MIA, which indicates the direction of transmitral inflow and reflects the degree of restriction of mitral valve opening (Figure 6). In patients with functional MR, subvalvular tethering reduces the leaflet mobility even during diastole, especially the excursion of the anterior leaflet. In the RMAP procedure, posterior leaflet tethering is augmented by the anterior displacement of the posterior mitral annulus, with persistent restriction of the anterior leaflet excursion. The combination of the persistent restriction of anterior leaflet excursion and the augmented posterior leaflet tethering diminishes the mitral inflow angle, changing the inflow direction posteriorly. An additional posterior PMR procedure not only relocates the papillary muscle, but also pushes the posterior mitral annulus down towards the apex. This induces a mitral annular tilt effect, which involves augmentation of posterior tethering, diminution of anterior leaflet excursion, decreased leaflet tip opening distance, and a comprehensively severe reduction in MIA, reflecting severe impairment of diastolic mitral inflow. On the other hand, an additional anterior PMR procedure not only compensates for the posterior leaflet tethering induced by RMAP, but also improves the restricted diastolic excursion of the anterior leaflet. However, the relocation suture can limit the maximal excursion of the anterior leaflet, which may result in a reduced distance between the open leaflet tips. As a comprehensive effect of those factors, MIA is well preserved after anterior PMR, and the diastolic mitral valve configuration is maintained most physiologically among these 3 surgical procedures.

### Clinical implications

To reduce persistent/recurrent MR after mitral valve repair for functional MR, PMR in conjunction with RMAP is an effective procedure, and one we believe is safe and reproducible. In previous reports, although functional MS after RMAP has been reported [23]-[25], the influence of PMR on diastolic mitral valve opening has not been emphasized. This study suggests that the direction of papillary muscle relocation influences the mitral valve configuration and function. An anterior PMR procedure could produce a more physiological mitral valve configuration after mitral valve repair, improving both systolic and diastolic subvalvular tethering. It can be expected to achieve long-term durable mitral valve repair, avoiding functional MS. The new index, MIA, could be useful for a comprehensive evaluation of the degree of mitral valve opening during diastole.

### Study limitations

This study has several limitations. It is a retrospective review of data and the number of patients is relatively small. Second, the follow-up term was relatively short. The long-term fate of the relocated papillary muscles is unknown. Over time, the papillary muscles may stretch to overcome the stress created upon them by the sutures. Long-term follow-up should be pursued to evaluate the presence of recurrent MR. Third, the study population consisted of patients with different etiologies of functional MR. However, we believe that the influence of surgical procedural differences on mitral valve configuration is equivalent regardless of the etiology. Last, diastolic mitral leaflet excursion was not evaluated by exercise stress echocardiography. Postoperative transthoracic echocardiography at rest tended to reveal a higher transmitral pressure gradient in the posterior PMR group than in the other groups, but the difference did not reach statistical significance (data not shown). Functional MS caused by diastolic subvalvular tethering can be dynamic, with significant exercise-induced deterioration. The assessment of diastolic mitral valve area and transmitral pressure gradient by exercise stress echocardiography could precisely demonstrate the dynamic nature of functional MS. If a correlation between MIA at rest and functional MS during exercise could be established by further study, the degree of functional MS might be deduced from an evaluation of MIA, without the need for exercise stress echocardiography.

## Conclusions

In the surgical treatment of functional MR, a PMR procedure in addition to RMAP was effective in reducing systolic MR. However, mitral valve opening assessed by MIA was restricted even after RMAP alone. The restriction was severely augmented after additional posterior PMR, but was attenuated after additional anterior PMR. The papillary muscles should be relocated in the direction of the anterior annulus to preserve the diastolic opening of the mitral valve.

## Authors’ original submitted files for images

Below are the links to the authors’ original submitted files for images. Authors’ original file for figure 1 Authors’ original file for figure 2 Authors’ original file for figure 3 Authors’ original file for figure 4 Authors’ original file for figure 5 Authors’ original file for figure 6

## Abbreviations

MR: Mitral regurgitation
RMAP: Restrictive mitral annuloplasty
PMR: Papillary muscle relocation
LV: Left ventricle
MIA: Mitral inflow angle
ANOVA: Analysis of variance
MS: Mitral stenosis
