# Supplementary figures and images for: Influence of procedural differences on mitral valve configuration after surgical repair for functional mitral regurgitation: in which direction should the papillary muscle be relocated?
Source: J Cardiothorac Surg. 2014 Dec 10;9:185. doi: 10.1186/s13019-014-0185-6 (PMC4272782; doi:10.1186/s13019-014-0185-6)

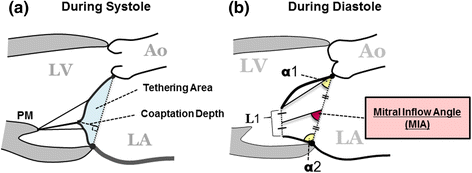

Supplement: Supplementary file 1 — Authors’ original file for figure 1 [file 13019_2014_185_MOESM1_ESM.gif]

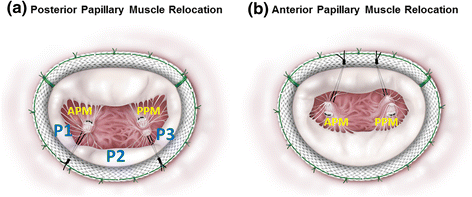

Supplement: Supplementary file 2 — Authors’ original file for figure 2 [file 13019_2014_185_MOESM2_ESM.gif]

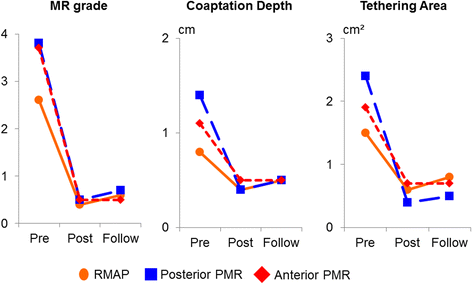

Supplement: Supplementary file 3 — Authors’ original file for figure 3 [file 13019_2014_185_MOESM3_ESM.gif]

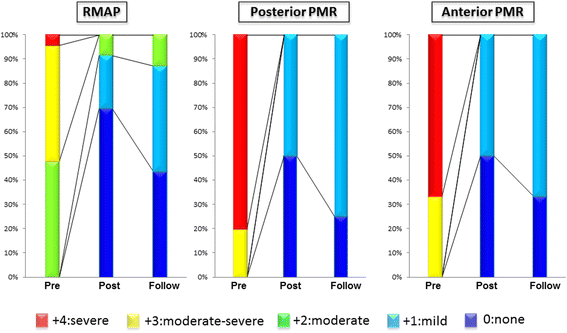

Supplement: Supplementary file 4 — Authors’ original file for figure 4 [file 13019_2014_185_MOESM4_ESM.gif]

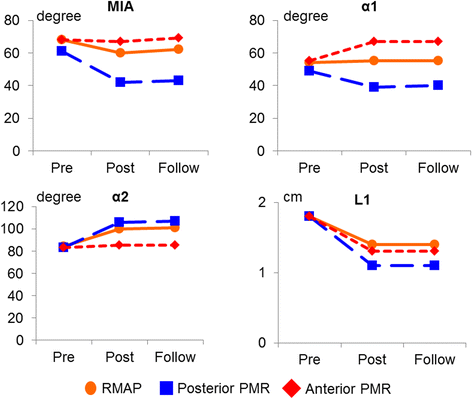

Supplement: Supplementary file 5 — Authors’ original file for figure 5 [file 13019_2014_185_MOESM5_ESM.gif]

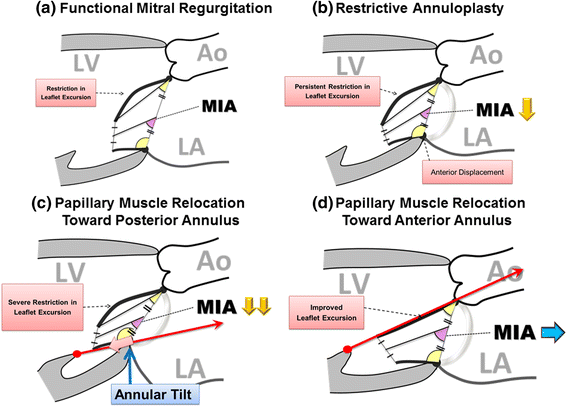

Supplement: Supplementary file 6 — Authors’ original file for figure 6 [file 13019_2014_185_MOESM6_ESM.gif]
